# An Exploratory Evaluation of the Present Prevalence of Hepatitis D Infection in Selected Populations from Venezuela, Contrasted with Past Studies in the Country

**DOI:** 10.3390/microorganisms14081788

**Published:** 2026-08-14

**Authors:** María Zulay Sulbarán, Julie Andreina Beltrán, Angélica Marín, Iría Lozano, Edgardo Mengual, Carolina Castejón, Jeanyberth Cobis, Andrea Lozano, Mario Comegna, Rosalía Perazzo, Mercedes De Sousa, Nahír Martínez-Urbina, Mariana Hidalgo, Yoneira Fabiola Sulbarán, Marwan Aguilar, Martha Bravo, Isabelle Chemin, Rossana Celeste Jaspe, Flor Helene Pujol

**Affiliations:** 1 Laboratorio de Virología Molecular, Centro de Microbiología y Biología Celular, Instituto Venezolano de Investigaciones Científicas, Caracas 1020, Venezuela; zulaysulbaran26@gmail.com (M.Z.S.); beltranjulie@gmail.com (J.A.B.); angelicamarin1508@gmail.com (A.M.); yfsulbara@gmail.com (Y.F.S.); 2 Unidad de Microscopía Electrónica, Centro de Química, Instituto Venezolano de Investigaciones Científicas, Apdo. 20632, Caracas 1020, Venezuela; 3 Unidad Médica de Investigación “Dr. Shubert Camacho”, Facultad de Medicina, Universidad del Zulia, Maracaibo 4001, Venezuela; iriacamacho@hotmail.com (I.L.); drmengual1@gmail.com (E.M.); carocastejon19@gmail.com (C.C.); paolacobisc@gmail.com (J.C.); av.trabajando@gmail.com (A.L.); 4 Dolphin Medical Research, BioSouthTech Inc., Sweetwater, FL 33172, USA; 5 Instituto de Investigaciones Biológicas “Doctores Orlando Castejón y Haydee V Viloria de Castejón”, Facultad de Medicina, Universidad del Zulia, Maracaibo 4001, Venezuela; 6 Servicio de Infectología, Hospital Vargas, Caracas 1070, Venezuela; mcomegna20076@gmail.com; 7 Sección de Hepatología, Hospital Miguel Pérez Carreño, Caracas 1010, Venezuela; hepatitiscvenezuela@gmail.com; 8 Servicio de Gastroenterología, Hospital Vargas, Caracas 1070, Venezuela; mdesousarincon@gmail.com; 9 Sección de Virología, Instituto de Medicina Tropical, UCV, Caracas 1053, Venezuela; nahirmu@gmail.com; 10 Laboratorio de Inmunoparasitología, Centro de Microbiología y Biología Celular, Instituto Venezolano de Investigaciones Científicas, Caracas 1020, Venezuela; mariana.hidalgo.r@gmail.com; 11 Instituto Nacional de Higiene “Rafael Rangel”, Caracas 1053, Venezuela; marwaguilar@gmail.com (M.A.); martharbravo@hotmail.com (M.B.); 12 UMR 1350, The Lyon Hepatology Institute (IHU EVEREST), F-69004 Lyon, France

**Keywords:** hepatitis D virus, hepatitis B virus, genotype, fulminant hepatitis, indigenous populations

## Abstract

Hepatitis D virus (HDV) is a satellite virus that requires the presence of a helper virus, the hepatitis B virus (HBV). Soon after its discovery by Mario Rizzetto in 1977, severe outbreaks of fulminant hepatitis D occurred in the past century, in indigenous populations from the Western and Southern (Amazon) regions of Venezuela, while the indigenous population from the Eastern region (Orinoco Delta) has been found to be free of this virus. Data on the prevalence of HDV infection in Venezuela are still highly imprecise. The aim of this study is to provide insights into the current situation of this disease in the country. Evidence of exposure and infection with HDV was found again mainly in indigenous populations, although also one patient with hepatitis B from the capital was found to be infected with HDV genotype 1. The genotype found in the indigenous Bari population was HDV genotype 1, the same lineage (a putative new subgenotype 1j) previously identified in this region in both Venezuela and Colombia. Although scarce information is still available about the burden of HDV infection in Venezuela, the few studies available are consistent in showing an epidemic concentrated mainly in some, but not all, indigenous populations. Reflex testing is needed to have a better appraisal of this disease in the country.

## 1. Introduction

Hepatitis D virus (HDV) is a satellite virus, a member of the *Kolmioviridae* family, that requires the presence of a helper virus, the hepatitis B virus (HBV), for its replication, exclusively for enveloping its virion and infecting susceptible cells [1]. Infection with HDV is frequently associated with a more severe disease than HBV mono-infection: it may present with fulminant hepatitis or a more rapid progression to cirrhosis and hepatocellular carcinoma [1].

Soon after its discovery by Mario Rizzetto in 1977 [2], a severe outbreak of fulminant hepatitis occurred in Venezuela [3]. HDV infection was found co-infecting patients with HBV. These findings stimulated a retrospective investigation of severe hepatitis outbreaks in Colombia (Santa Marta fever) and Brazil (Labrea fever), allowing the identification of HDV co-infecting with HBV in these patients [4,5,6].

**Figure 1:**
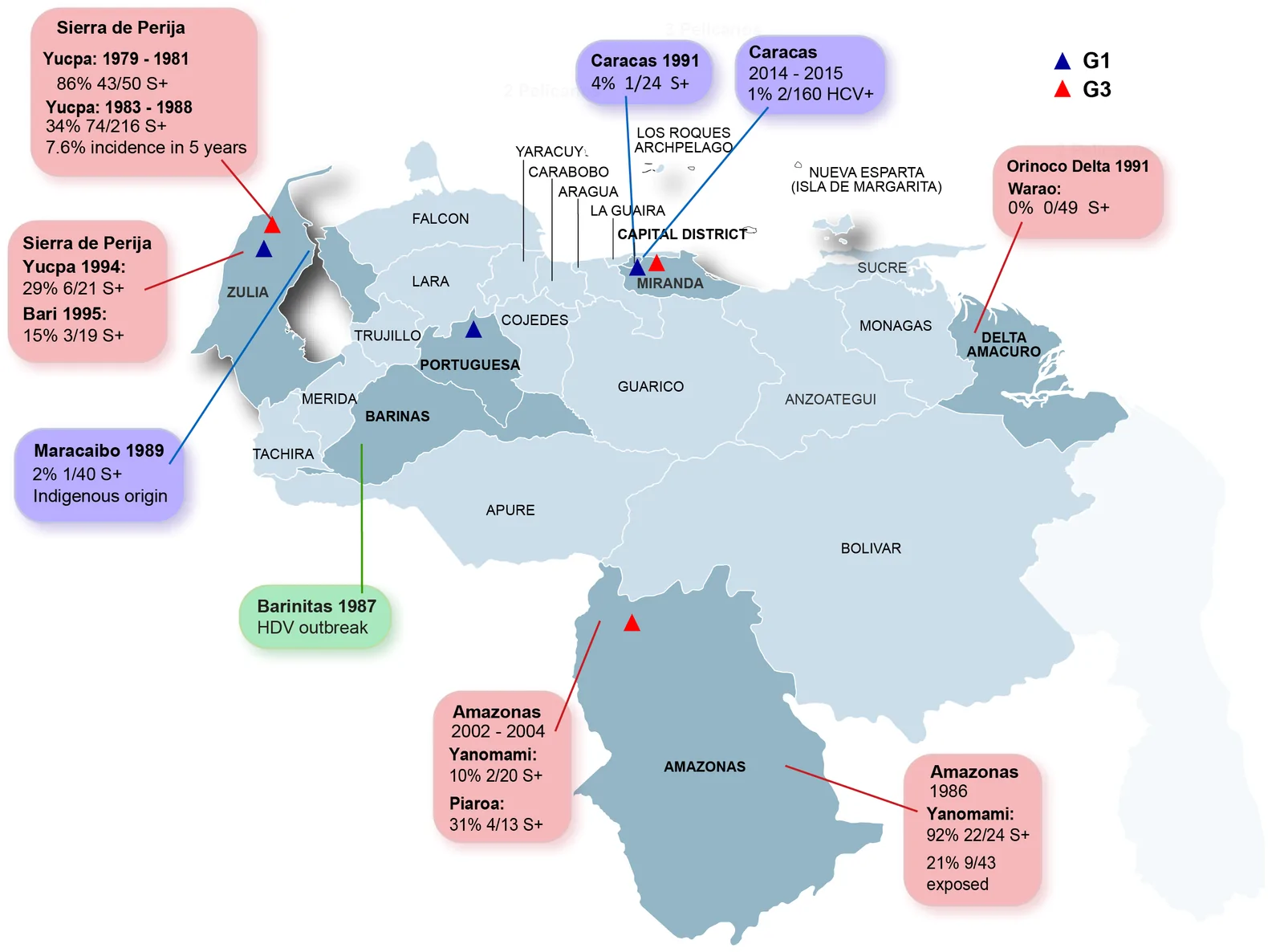
HDV infection in Venezuela from 1979 to 2015. Literature available at PubMed and Scielo, using the terms “hepatitis D” or “hepatitis delta” and “Venezuela”, including the prevalence of anti-HDAg (anti-HDV antigen) or HDV RNA, was analyzed for this study. Most of the seroprevalence studies were performed on HBsAg-positive (S+) individuals, except for the protracted outbreak of 1986 in the Amazon, where individuals with antibodies to HBV core were also tested (exposed), and the study of HCV-infected individuals of 2014–2015 in Caracas. The pink, lilac and green boxes refer to indigenous, urban, and rural populations, respectively. HDV genotypes 1 and 3 (blue and red triangles, respectively) have been found in the country [3,7,8,9,10,11,12,13,14].

During the severe outbreak of fulminant hepatitis of 1979–1981 in Western Venezuela (Sierra de Perijá), 149 individuals from the Yucpa indigenous group developed hepatitis; 34 persons died and at least 22 developed chronic hepatitis [3]. The most affected individuals were children and young adults. Most patients had evidence of HDV superinfection; 86% of Hepatitis B surface antigen (HBsAg)-positive patients had antibodies against HDV (anti-HDV), and the viral antigen was found in 7 of 9 autopsies. Serological data showed that most infections were due to HDV superinfection of hepatitis B carriers, and that more than 60% of these infections progressed to chronic disease [3]. A follow-up of 5 years of this epidemic (1983–1988) showed an incidence of HDV infection of 7.6% [7]. In addition to the Yucpa indigenous group from Western Venezuela, a high HDV infection rate was found in the Bari group, which also lives in Sierra de Perijá [8] (Figure 1).

The discovery of HDV circulation among indigenous groups prompted the investigation of other indigenous groups in Venezuela, such as the Yanomami from the Amazon Basin in Southern Venezuela, where a protracted HDV outbreak was documented [9]. In addition to Yanomami, HDV was also found in another indigenous group from the Venezuelan Amazon Basin, the Piaroa (Figure 1) [10]. In contrast, in the Warao indigenous group of the Orinoco Delta (Eastern Venezuela), although with a significant prevalence of HBV exposure, no HDV infection was documented (Figure 1) [11].

Despite the high prevalence of HDV infection found in many indigenous groups in Venezuela, HDV prevalence seemed to be low in urban settings, as shown in the few studies performed in Maracaibo and Caracas [7,8]. A cluster of high prevalence of HBV exposure with a high prevalence of HDV co-infection was described in 1987 in the rural locality of Barinitas, in the Barinas State [9,10]. These findings prompted the initiation of a HBV vaccination program in the locality, and a clear reduction in HBV exposure was observed over the following 20 years [10]. More recently (2014–2015), in a study of 160 viremic HCV-infected patients from Caracas, anti-HDV antibodies were detected in two patients and HDV RNA in one of these two sera, without any HBV serological marker [11] (Figure 1).

This virus displays a high variability in its small RNA genome (around 1700 nt) [1]. Two genotypes were originally described for HDV. A decade after documenting the severe forms of hepatitis in the Amazon, a divergent third genotype of HDV was discovered in the region, circulating with HBV genotype F [15]. Since then, five other genotypes have been described, most of them from Africa [16]. Several subgenotypes have also been proposed within the 8 genotypes of HDV. Particularly, for HDV genotype 1, up to 9 subgenotypes have been defined [17].

Both HDV genotypes 1 and 3 have been found circulating in Venezuela, in Bari and Yucpa indigenous populations from the Sierra de Perijá [11,12], while only genotype 1 has been found in Caracas and Caño Delgadito (rural setting) [11,12]. In contrast, only HDV genotype 3 was identified among the Yanomami indigenous populations in the Amazon [11] (Figure 1). However, the number of sequences in these studies is too low to draw any significant conclusion regarding HDV diversity in Venezuela.

The global prevalence of HDV infection remains unclear [1]. For example, in the same year, two reports showed divergent worldwide estimates: 12 million [18] vs. 48–60 million [19]. The situation is even more approximate for Venezuela. In the first study, Venezuela appears as “no data” [18] and in the second, the country appears because of the historical documentation of HDV cases in indigenous populations [19]. The aim of this study is to provide some clues on the present situation of this disease in selected populations in Venezuela.

## 2. Materials and Methods

### 2.1. Population Groups

This study was approved by the Human Bioethical Committee of the Venezuelan Institute for Scientific Research (IVIC) under the ethical approval codes CD-2024/0874, 2024PG445, and CBE-UNIMED-04-2023. Blood samples were obtained after written informed consent from different population groups: patients with HBV (monoinfected *n* = 60), HCV infection (monoinfected *n* = 145), collected between 2023 and 2026, and people living with HIV (PLWH, *n* = 129), collected between 2022 and 2025, most of whom were from Caracas. In addition, HBV-positive sera from indigenous (Bari) populations (HBsAg and/or anti-HBc positive), collected in 2023 from Sierra de Perijá, Municipio Machiques, Zulia state (Western Venezuela) (*n* = 53), were also available. The inclusion criteria for this study were individuals infected with any of the viruses mentioned and willing to participate. The selection criteria were:

- infection with HBV, because of the known necessity of HBV co-infection for HDV infection [1],
- infection with HCV, because of previous reports of HDV infection or exposure in HCV-infected patients [11,20,21],
- people living with HIV-1, because of the shared risk factors [18,22,23,24].

### 2.2. Prevalence of Anti-HDV Antibodies

Anti-HDV antibodies were detected using the anti-HDV ELISA test DIA Source (Louvain-la-Neuve, Belgium), which is a competitive ELISA. A total of 100 µL of sera was added to the wells. After incubation at 37 °C for 60 min and washing, 100 µL of enzyme conjugate were added. After repeating the same incubation and washing procedure, 100 µL of TMB/H_2_O_2_ substrate were added and the microplate was incubated at room temperature for 20 min. Then 100 µL of 0.3 M sulfuric acid were added into all the wells. The absorbance was measured at 450 nm with a background subtraction at 620–630 nm. Samples with absorbance values below the value (negative control + positive control)/5) were considered positive.

### 2.3. Detection of HDV RNA and HBV DNA, and Amplification of the Complete HDV Genome

Total RNA was extracted from sera using the QIAamp Viral RNA Mini Kit (QIAGEN GmbH, Hilden, Germany). HDV RNA was detected by RT-nested PCR of the HDV antigen region, using primers 5413-8276m for the first round and 5414-5415 for the second one, as previously described [12]. The complete HDV genome was amplified by RT-PCR using the “Superscript III One-Step RT-PCR system” (Thermo Fisher Scientific, Waltham, MA, USA) to obtain two amplicons that encompass the whole genome, using primer pairs 320s/8276 for fragment 1 modified (F1m) and 900s/503as for fragment 2 (F2). Subsequently, hemi-nested PCR was carried out with “Platinum Taq DNA Polymerase” (Thermofisher Scientific, Waltham, MA, USA) with primer pairs 320s/5415 for fragment 1 modified (F1m_2) and 900s/440as for fragment 2 (F2_2) for the second one according to the protocol suggested by Angelice et al. [25].

HBV DNA was detected by real-time PCR, using primers preSf-preSr2 and preSprobe from the preS genomic region of HBV [12]. For sequencing, amplicons were generated by nested PCR of the S region using primers 58P-1100N for the first round and S6-S3as for the second round, as previously described [26]. The different primers and PCR conditions are listed in Table 1.

### 2.4. Sequencing

Amplicons (*n* = 5) were sequenced using the automatic sequencing service of Macrogen Inc. (Seoul, Republic of Korea).

Complete genome sequencing was performed by next-generation sequencing. Libraries were prepared using the Illumina DNAPrep Kit (Illumina, San Diego, CA, USA), using the two genomic fragments for each three complete HDV genomes as input DNA, as described in the previous section. Libraries were pooled, quantified (Qubit DNA HS, Thermo Scientific, Waltham, MA, USA), and sequenced on a MiSeq 100 platform using a 300-cycle V2 kit (Illumina, San Diego, CA, USA) with paired-end sequencing at a loading concentration of 50 pM. Viral genome assembly was performed using the Genome Detective Virus Tool (https://www.genomedetective.com/, accessed on 6 June 2026) [27].

The nucleotide sequences have been deposited in the GenBank database under the accession numbers PZ653092-PZ653096 (HDV) and PZ668057-PZ668062 (HBV).

#### Phylogenetic Analysis

Sequence alignments were performed using MAFFT [28]. Phylogenetic trees were inferred by the Maximum Likelihood method, using IQ-Tree [29] and edited using ITol version 7.4.2 [30]. Bootstrap values over 80 were considered to indicate a highly supported clade. Genetic distances were assessed using MEGA version 12 [31].

## 3. Results

A total of 308 sera were tested for anti-HDV antibodies and 290 for HDV RNA, and evidence of infection and/or exposure was found in 11 of them (Table 2). No anti-HDV antibodies nor HDV RNA were found in HCV-infected patients from Caracas. Neither anti-HDV antibodies nor HDV RNA were found in PLWH, even with HBV or HCV co-infection. Anti-HDV antibodies, without viremia, were found in two sera from indigenous individuals: a Yucpa living in Maracaibo and in 8 Bari individuals, with or without viremia. HDV RNA was found in one HBV-infected patient from Caracas and in 4 sera from Bari indigenous individuals. Anti-HDV could not be tested for one of the Bari sera with viremia, and another was negative; thus, the prevalence of HDV exposure (anti-HDV and/or HDV RNA) was 10/53 (19%). Neither HDV RNA was found in HBV nor HCV-infected patients from other states of Venezuela (Table 2).

**Figure 2:**
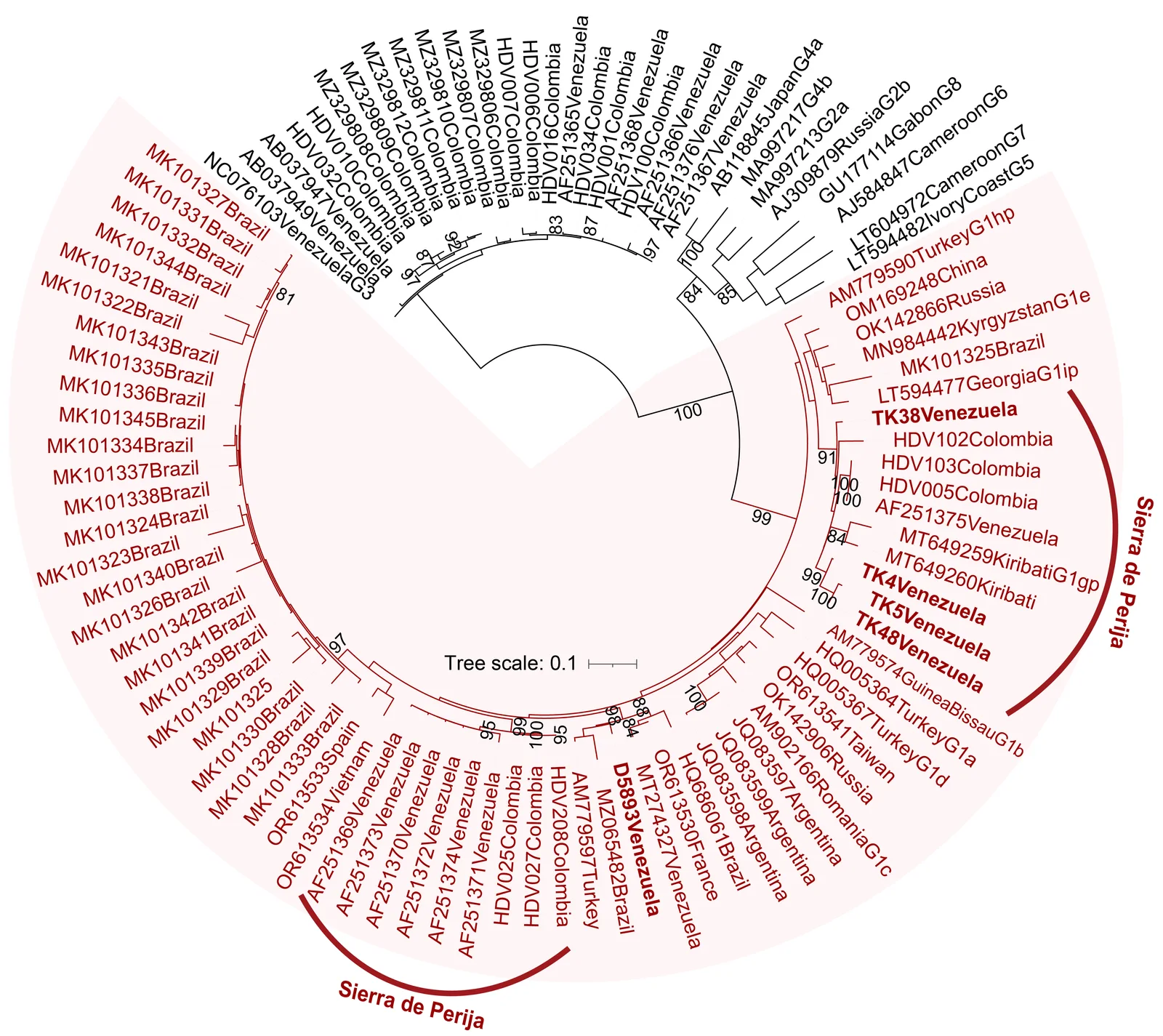
Phylogenetic tree of the HDAg genomic region (332 nt). Maximum likelihood tree, using the TPM3u+F+G4 model, and 1000 bootstrap replicates. Bootstrap values over 80 are shown. The sequences are named after their accession numbers, the country when known, and the genotype/subgenotype for reference sequences [17]. HDV genotype 1 sequences are shown in red, and the five sequences from this study are shown in bold. Sierra de Perija: location of the Bari indigenous communities.

For the five sera positive for HDV RNA, complete genome sequences were obtained for three, and a partial sequence (HDAg genomic region) for the two others. All the HDV isolates belonged to HDV genotype 1 (Figure 2). Phylogenetic analysis of the HDAg region showed that the isolates from indigenous Bari (TK, *n* = 4) grouped closely with sequences obtained in previous studies, from indigenous populations of the same region (Sierra de Perijá), in Venezuela and Colombia (Figure 2). In these studies, two clades of HDV genotype 1 have been reported [12,32] (Figure 2). The sequences from this study were grouped only into one of these two clades (Figure 2). Other sequences of HDV genotype 1 from Argentina and Brazil (the only countries in South America for which HDV genotype 1 is available at GenBank) were not grouped with either of these two lineages. The HDV sequence from the patient from Caracas (D5893) was grouped with sequences from France, Turkey, and a sequence from a Venezuelan patient co-infected with HCV from a previous study [11].

**Figure 3:**
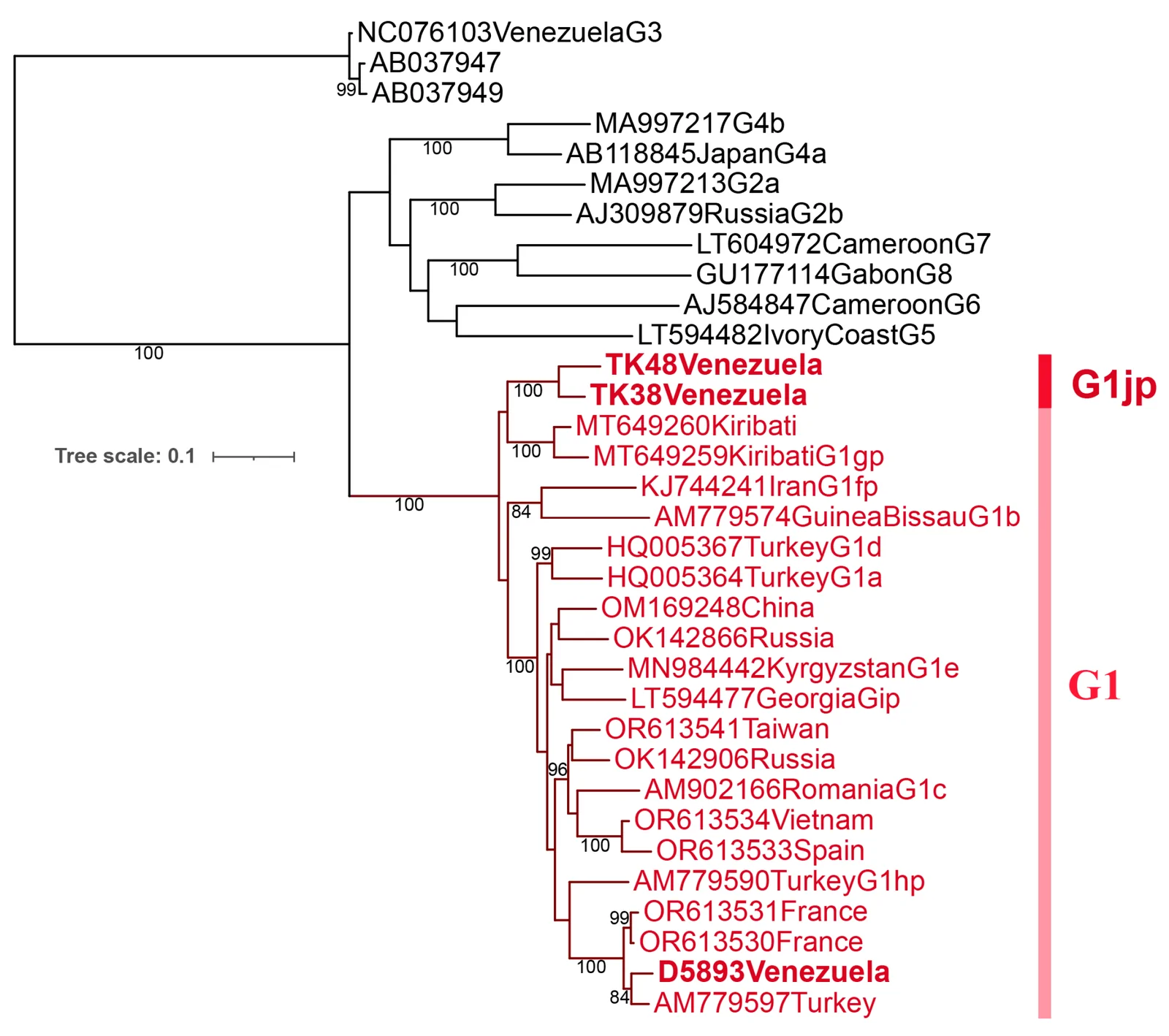
Phylogenetic tree of the HDV complete genome. Maximum likelihood tree, using the TVM+F+I+R3 model, and 1000 bootstrap replicates. Bootstrap values over 80 are shown. The sequences are named after their accession numbers, the country when known, and the genotype/subgenotype for reference sequences [17]. HDV genotype 1 sequences are shown in red, and the three sequences from this study are shown in bold. G1jp: putative G1j subgenotype.

Phylogenetic analysis of the three complete genome sequences, two from Bari indigenous sera and one from a patient from Caracas, is shown in Figure 3. This sequence was grouped with sequences from France and Turkey. Complete genome sequences of the two Bari isolates were grouped with two sequences from Kiribati (the most similar sequences available at GenBank), but in a different clade, with a mean identity of 83%, below the threshold accepted for genotype 1 subgenotypes [16] (Figure 3).

**Figure 4:**
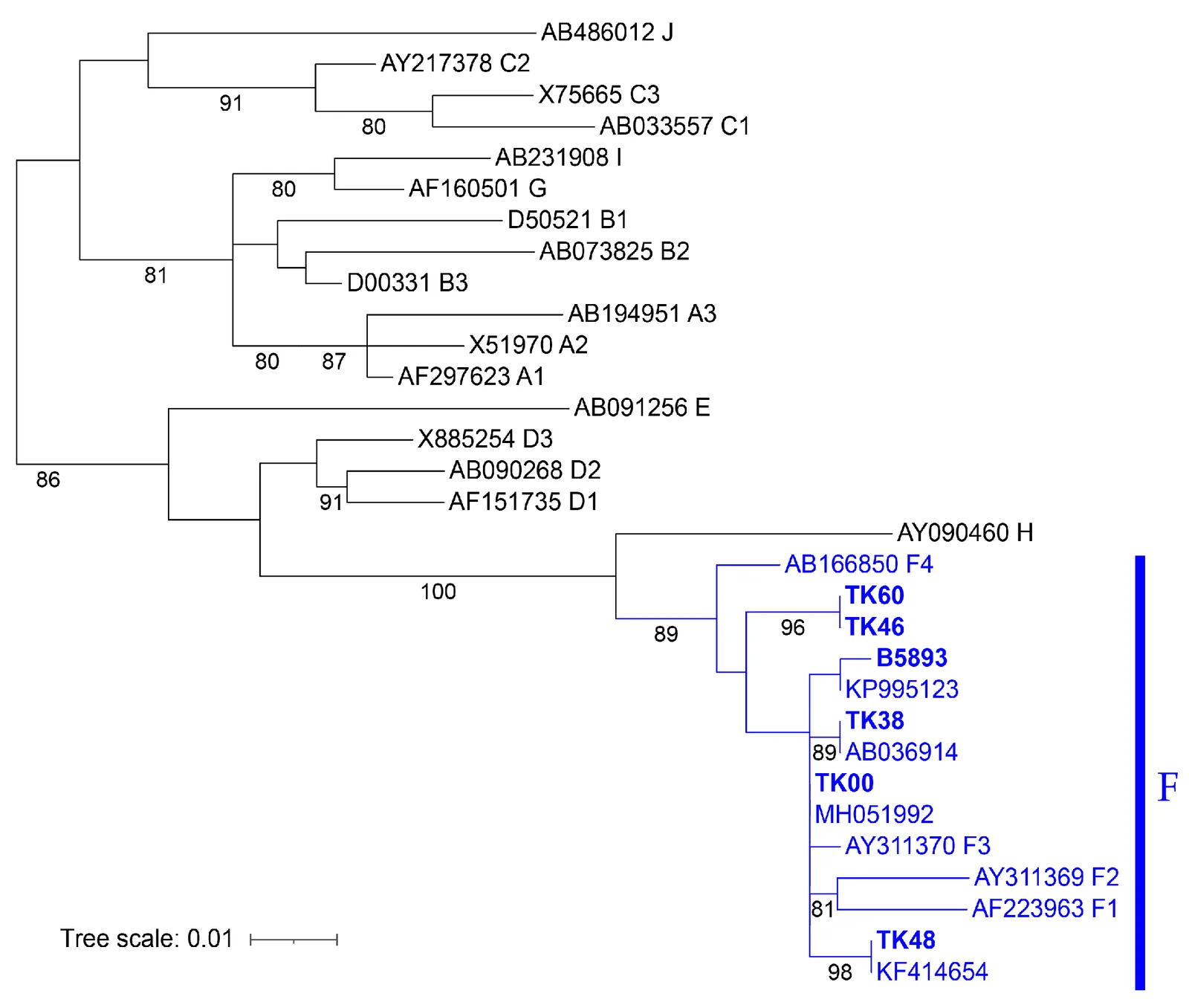
Phylogenetic tree of the HBsAg partial gene isolates (299 nt). Maximum likelihood tree, using the TPM2+G4 model, and 1000 bootstrap replicates. The sequences are named after their accession numbers and the genotype/subgenotype for reference sequences [26]. HBV genotype F sequences are shown in blue, and the 6 sequences from this study in bold.

A small fragment of the sequence of the HBV S region was available for five isolates from the Bari indigenous population (two co-infected with HDV) and from the HDV co-infected patient from Caracas: all the HBV isolates belonged to HBV genotype F, probably subgenotype F3, the most common in Venezuela, although the small size of the sequenced fragment did not allow for a proper discrimination between subgenotypes (Figure 4).

No anti-HDV antibodies were detected in the sera of two HDV-viremic samples from the Bari population (Table 3). Patient 5893 is a female patient with a fibrosis grade 4.

## 4. Discussion

Even if scarce information is available about the burden of HDV infection in Venezuela, the few studies available are consistent in showing an epidemic concentrated mainly in some, but not all, indigenous populations. As described previously in Venezuela, while a high HBV prevalence is found among all the indigenous population groups, HDV co-infection is not always found among these groups, as shown for the Warao indigenous population from the Orinoco Delta. Similarly, in the Arctic, HDV co-infection has been described in Greenland [33,34] but not in Alaska [35]. However, HDV genotypes have not been described in any of these studies [36]. HDV infection is believed to be infrequent among other Aboriginal North American populations [37].

After the discovery of HDV circulation in indigenous populations in Venezuela, several HBV vaccination programs were undertaken, particularly in the different indigenous populations of Venezuela [8,38,39]. A clear reduction in the prevalence of active HBV infection and exposure has been documented in populations subjected to vaccination [8,10,39]. Universal HBV vaccination at birth was introduced in 1998. Following initial poor coverage, it reached 87% in 2015. However, lower and oscillating vaccination coverage has been observed since then, with maximum values reaching around 60% [40]. Therefore, apparently, HBV vaccination may only partially explain the relatively low number of HDV positive cases among the non-indigenous Venezuelan population. Some preliminary evidence suggests a decrease in HBV prevalence in recent decades: a reduction in morbidity and mortality associated with HBV infection and estimated prevalence has been observed [41,42]. More studies are needed to evaluate the real burden of HBV infection in Venezuela, to then analyze the burden of HDV infection.

HDV infection has been found to increase in HBV-infected individuals living with HIV [18,22,23,24]. No evidence of HDV exposure was found among PLWH, at least in the capital city of Caracas. Although still controversial, evidence of exposure to or HDV infection in HCV-infected patients has been documented in three previous studies, with the apparent absence of HBV infection [11,20,21]. In this study, none of the anti-HCV positive sera were found positive for anti-HDV antibodies or for HDV RNA. More studies are needed to define the exact role of HCV infection in HDV co-infection, without discarding the presence of a silent occult HBV infection [1]. Only one case of HDV infection was found among a non-indigenous HBV co-infected patient. The HDV sequence of the HBV co-infected patient from Caracas was grouped with a clade that includes the sequence of the HDV sequence found in a Venezuelan patient co-infected with HCV in a previous study [11], suggesting that this clade might be circulating, although at low prevalence, in the country. Again, the identification of HDV infection in the capital city suggests that HDV should not be considered an exclusively indigenous rural disease.

A high prevalence of HDV exposure was found among the Bari indigenous population, either anti-HDV or HDV RNA. In addition, in two cases, HDV RNA was detected without anti-HDV antibodies, indicating either an acute or serologically occult HDV infection. HDV RNA without anti-HDV antibodies has also been found in Argentinean HBV-infected patients: the authors propose a synchronous testing of anti-HDV antibodies and HDV RNA for a better appraisal of HDV infection [43].

HDV infection is highly frequent in the Western Amazon region of South America, with predominant circulation of HDV genotype 3: this region includes indigenous populations from Brazil [25,44], Colombia [32], Ecuador [45], Peru [11] and Venezuela [12]. Circulation of HDV genotype 1 has also been reported more recently in the Amazon [32] and in Brazil, although in the latter country, in non-indigenous populations [46]. In addition to HDV genotype 3, which is expected in indigenous populations [47], HDV genotype 1 is also present in the Bari population of Western Venezuela. This genotype was already identified in these indigenous groups in Venezuela [12] and Colombia [17]. Contacts with Europeans or slaves’ escape to the Sierra de Perijá (Western Venezuela, at the frontier with Colombia) have been documented in the past centuries [48], and these contacts might have been responsible for the introduction of HDV genotype 1 in these populations.

However, HDV genotype 1 was not identified in the samples sequenced from the outbreak of fulminant hepatitis between 1979 and 1981 [13]. Two reasons may explain the lack of detection of HDV genotype 1 in this previous study: a later introduction of the foreign HDV genotype 1 in these populations, or a bias in the few (3) samples analyzed in this first study, since all the isolates were from individuals with a severe presentation of the disease, which is more frequent in infection with HDV genotype 3 [1]. HDV infection has also been described in indigenous individuals from Argentina, infected with HDV genotype 1 [49], the only genotype described in this country to date. The HDV genotype 1 isolates identified in the indigenous populations of Western Venezuela and Northern East of Colombia were grouped into two clades, but neither the Brazilian nor Argentinian sequences were related to these isolates.

HDV genotypes are classified into genotypes according to an intergenotype divergence of 80% in the complete genome sequence, and further classified into subgenotypes based on an inter-subgenotype similarity of >90% for seven genotypes and >84% for HDV-1 [16]. The complete genome sequences of the two HDV isolates circulating in the Bari population group displayed less than 84% similarity with the closest isolates belonging to the newly proposed putative subgenotype 1g from Kiribati [17], and with the other HDV genotype 1 sequences, suggesting the presence of a new putative subgenotype 1j.

This study has several limitations: the small number of HBV-positive sera tested to appraise the real prevalence of HDV infection, and it was limited to a population mostly from the capital city and one indigenous population group. Studies are warranted in other settings in Venezuela, particularly in indigenous populations, to assess the real burden of HDV in the country.

## 5. Conclusions

This preliminary study suggests that HDV remains a concern in Venezuela, particularly among indigenous populations. As in many other countries [50], many gaps remain concerning the prevalence of HDV and also HBV infection in Venezuela. The evaluation of indigenous individuals allowed us to suggest that HDV is still active and some viral variants different from the expected HDV genotype 3 are circulating, with the identification of a new putative genotype 1j. Reflex testing, and eventually synchronous testing, of HBV-positive cases [24], as well as HCV-positive ones in some cases, is warranted to assess the real burden of this disease in Venezuela.

## Figures and Tables

**Table 1:** Primers and PCR conditions used for amplification of the HBV and HDV genomes [12,25,26].

Genomic Target	Primers	Sequence	PCR Conditions
HDAg	First round		55 °C 20 min, 95 °C 5 min
	5413	5′-GCCCAGGTCGGACCGCGAGGAGGT-3′	44 cycles: 95 °C 20 s, 57 °C 20 s, 72 °C 20 s
	8276m	5′-ACAAGGAGAGGCAGGAYCACMGAMGAA-3′	72 °C 5 min
	Second round		95 °C 5 min
	5414	5′-GAGATGCCATGCCGACCCGAAGAG-3′	40 cycles: 95 °C 20 s, 57 °C 20 s, 72 °C 20 s
	5415	5′-GAAGGAAGGCCCTCGAGAACAAGA-3′	72 °C 5 min
HDV complete genome	F1m		First round F1m and F2
	320s	5′- CCAGAGGACCCCTTCAGCGAAC-3′	55 °C 20 min, 95 °C 5 min
	8276m	5′-ACAAGGAGAGGCAGGAYCACMGAMGAA-3′	44 cycles: 95 °C 20 s
	F2		57 °C 20 s, 72 °C 95 s
	900s	5′-CATGCCGACCCGAAGAGGAAAG-3′	72 °C 5 min
	503as	5′-CCCCGGGATAAGCCTCACTCG-3′	
	F1m_2		Second round F1m_2
	320s	5′-CCAGAGGACCCCTTCAGCGAAC-3′	and F2_2
	5415	5′-GAAGGAAGGCCCTCGAGAACAAGA-3′	95 °C 5 min
	F2_2		40 cycles: 95 °C 20 s,
	900s	5′-CATGCCGACCCGAAGAGGAAAG-3′	57 °C 20 s, 72 °C 80 s
	440as	5′-CCAGAGGACCCCTTCAGCGAAC-3′	72 °C 5 min
HBV qPCR	preSfm	5′-GAATCCTCACAATACCRCAGAGT-3′	95 °C 10 min
	preSralt	5′-GGTTGGGGACTGCGAATTTTGGCC-3′	40 cycles: 97 °C 30 s, 54 °C 90 s, 32 °C 1 min
	preSprobe	5′(FAM)-CTAGACTCGTGGTGGACTT-(BHQ)3′	
HBsAg	First round		94 °C 3 min
	58P	5′-CCTGCTGGTGGCTCCAGTTC-3′	39 cycles: 94 °C 45 s, 45 °C 45 s, 72 °C 1 min
	1101N	5′-GAAAGGCCTTGTAAGTTGGCGAG-3′	72 °C 7 min
	Second round		95 °C 10 min
	S6	5′-GCACACCGGGATCCGAGGACTGGGGACCCT-3′	35 cycles: 95 °C 20 s, 55 °C 20 s, 72 °C 45 s
	S3as	5′-CCCCAAWACCAVATCATCCATATA-3′	72 °C 5 min

**Table 2:** Prevalence of serological markers in selected population groups from Venezuela.

Locality	Population Group	Anti-HDV	HDV RNA
Caracas	HBV+ ^1^	1/24	1/43
	HCV+ ^1^	0/100	0/131
	HIV+ HBV+ ^2^	0/79	0/15
	HIV+ HCV+ ^2^	0/17	0/12
	HIV+ ^2^	0/33	0/3
Zulia	Yucpa ^3^	2/2	0/2
	Bari HBV+ ^4^	8/53	4/53
Sucre	General population: HBV+ ^1^	-	0/5
	Hemodialysis patients: HBV+ ^1^	-	0/12
Other states ^5^	HCV+	-	0/14

^1^ HBV+ patients: HBsAg or anti-HBc positive patients with HBV DNA. HCV+ patients: anti-HCV positive patients with HCV RNA. ^2^ HIV+ HBV+ patients: HIV-1-positive patients with HBsAg or anti-HBc independently of HBV DNA status. HIV+ HCV+ patients: HIV-1-positive patients with anti-HCV, independently of HCV RNA status. HIV+: HIV-1 infected patients with no markers of HBV or HCV antigens or antibodies. ^3^ Two sera collected in Maracaibo city from indigenous Yucpa displaced females. ^4^ HBsAg and/or anti-HBc positive patients. ^5^ HCV positive patients from Carabobo (*n* = 7), Merida (*n* = 1), Sucre (*n* = 3) and Yaracuy (*n* = 3).

**Table 3:** HBV and HDV genotypes from isolates from the Caracas and Bari indigenous population.

Locality	Patient (Age in yr, and Sex)	HBV Genotype	Anti-HDV	HDV Genotype
Caracas	5893 (63, F)	F	+	1hp
Bari, Sierra de Perijá	TK0 (54, M)	F	-	-
	TK4 (42, F)	NA ^1^	-	1jp ^2^
	TK5 (48, M)	NA	-	1jp
	TK7 (40, M)	NA	+	-
	TK25 (55, M)	NA	+	-
	TK31 (47, F)	NA	+	-
	TK38 (45, M)	F	+	1jp
	TK46 (54, M)	F	+	-
	TK48 (34, M)	F	+	1jp
	TK60 (56, F)	F	-	-

^1^ Could not be amplified by PCR for sequencing. ^2^ Putative subgenotype 1j.

## Data Availability

The nucleotide sequences have been deposited in the GenBank database under accession number PZ653092-PZ653096 (HDV) and PZ668057-PZ668062 (HBV).

## Abbreviations

The following abbreviations are used in this manuscript:

Anti-HBc	Anti-HBV core positive
HBV	Hepatitis B virus
HBsAg	Hepatitis B surface antigen
HCV	Hepatitis C virus
HDV	Hepatitis D virus
HDAg	Hepatitis D antigen
